# TTLL4 mediates the PI3K/AKT/MDM2 pathway to promote hepatocellular carcinoma progression and predict patient prognosis

**DOI:** 10.1063/5.0267938

**Published:** 2025-06-30

**Authors:** Zeping He, Desheng Chen, Lei Li, Shanbao Li, Fangbin Song, Jinfeng Cai, Xueyan Guo, Yaohao Luo, Xinshuai Wang, Zeping Chen, Junming Xu

**Affiliations:** 1 Department of General Surgery, Shanghai General Hospital, School of Medicine, Shanghai Jiaotong University, Shanghai 200080, China; 2 Department of Hepatic Surgery and Liver Transplantation Center, Guangdong Provincial Key Laboratory of Liver Disease Research, The Third Affiliated Hospital of Sun Yat-sen University, Guangzhou 510630, China; 3 Department of Critical Care Medicine, The First Affiliated Hospital of Guangzhou Medical University, Guangzhou 510120, China

## Abstract

Hepatocellular carcinoma (HCC) is a highly lethal and heterogeneous tumor driven by the dysregulation of multiple genes. Tubulin tyrosine ligase-like 4 (TTLL4) has been linked to tumor progression, but its specific role in HCC pathogenesis remains unclear. RNA sequencing data, somatic mutation profiles, and clinical characteristics were analyzed from TCGA, GEO, and TIMER databases. The effects of TTLL4 on cell proliferation, migration, and apoptosis were studied using functional assays and flow cytometry. *In vivo*, tumor growth and metastasis were evaluated through subcutaneous implantation and tail vein injection. Immunohistochemistry assessed TTLL4 and Ki-67 expression. TTLL4 was upregulated in HCC and associated with poor prognosis, linking it to cancer progression and the PI3K–AKT signaling pathway. Knockdown of TTLL4 in HCC cells reduced proliferation, migration, and colony formation while increasing apoptosis. *In vivo*, TTLL4 knockdown slowed tumor growth and reduced lung metastasis. It also decreased the expression of proteins in the PI3K/AKT/MDM2 pathway, while overexpression upregulated these proteins. Rescue experiments further suggest that TTLL4 may exert its regulatory effects on this pathway by modulating PI3K expression levels. TTLL4 plays a significant role in HCC progression via the PI3K/AKT/MDM2 pathway and may serve as a novel therapeutic target for HCC diagnosis and treatment.

## INTRODUCTION

Hepatocellular carcinoma (HCC) is the predominant cause of cancer-related mortality globally.^1^ The prognosis of HCC remains poor, with a 3-year survival rate of only 12.7% and a median overall survival (mOS) of 9 months for end-stage patients.^2^ Epidemiological projections have suggested that the disease burden associated with HCC will persist at significantly elevated levels over the next decade. Notably, HCC patients consistently have poor prognoses, primarily due to high recurrence rates and the limited availability of efficacious therapeutic interventions. The malignant biological behavior of HCC can be influenced by oncogenic proteins, a diverse array of which are expressed in hepatoma cells. However, the specific tumorigenic mechanisms underlying these actions remain largely unknown.^3,4^ Given these challenges, it is of paramount importance to identify reliable biomarkers for both early detection and accurate prognosis. Furthermore, there is an urgent need to develop novel molecular-targeted therapeutic strategies for HCC that can effectively address the current limitations of treatment options.

The carboxy-terminal post-translational modifications (PTMs) of tubulin, including acetylation, detyrosination, tyrosination, polyglutamylation, and polyglycylation, are closely associated with cancer pathogenesis, particularly in cell death, chemotherapy sensitivity, migration, and invasion.^5,6^ Among these, tubulin tyrosine ligase-like 4 (TTLL4) is a key enzyme in tubulin glutamylation that adds single glutamate residues to multiple internal sites on the β-tubulin tail via γ-linkage. This modification leads to functional changes in tubulin, impacting crucial processes such as cell morphology, motility, division, intracellular transport, and secretion.^7–9^ Although its precise functions have not been extensively elucidated, TTLL4 has been implicated in the malignant progression of breast and pancreatic cancer.^10,11^ Notably, TTLL4 plays a significant role in pancreatic carcinogenesis through its polyglutamylase activity and subsequent modulation of chromatin remodeling. TTLL4-mediated polyglutamylation of PELP1 significantly alters its affinity for interaction with histone H3, thereby reducing the acetylation levels of histone H3. This modification is associated with the silencing of tumor suppressor genes and the development and progression of cancer.^11^ Furthermore, TTLL4-mediated monoglutamylation of cyclic GMP–AMP synthase (cGAS) at Glu302 inhibits its synthase activity, consequently attenuating the immune response to viral DNA infection. This mechanism may play a crucial role in the progression from hepatitis B to cirrhosis and HCC,^12^ suggesting its potential function as an HCC catalyst.

This investigation aimed to elucidate the role of TTLL4 in hepatocarcinogenesis by examining its expression pattern, assessing its clinical prognostic value, and investigating its oncogenic function and underlying mechanisms both *in vivo* and *in vitro*. Our findings demonstrated that TTLL4 was one of the two genes in the carboxy-terminal PTMs of tubulin that was highly expressed and correlated with poor prognosis in HCC. Notably, TTLL4 enhanced HCC proliferation, distant metastasis, and anti-apoptotic ability. The oncogenic effects of TTLL4 were potentially mediated through the PI3K/AKT/MDM2 signaling pathway. These results provided novel insights into the molecular mechanisms underlying HCC progression and offered potential avenues for developing targeted therapeutic strategies for this malignancy.

## RESULTS

### Upregulated TTLL4 expression in HCC

**FIG. 1.**
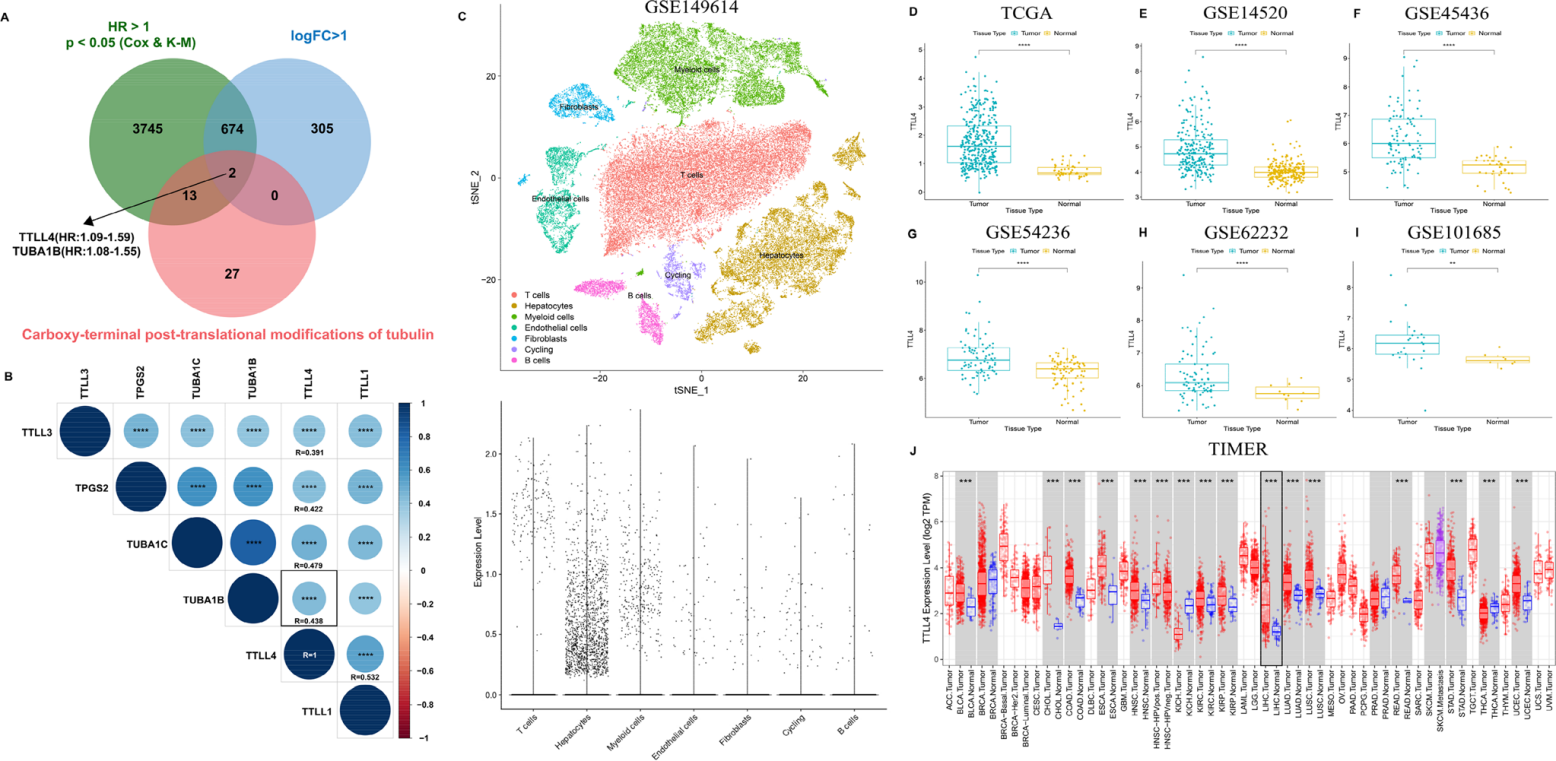
The expression of TTLL4 across several datasets. (a) The overlap among genes related to carboxy-terminal post-translational modifications of tubulin, differentially expressed genes, and prognostic genes from the TCGA liver cancer dataset. (b) Top five genes most correlated with TTLL4 expression in carboxy-terminal post-translational modifications of tubulin, analyzed by Pearson correlation. The expression of TTLL4 in (c) GSE149614, (d) TCGA cohort, (e) GSE14520, (f) GSE45436, (g) GSE54236, (h) GSE62232, and (i) GSE101685. (j) The expression of TTLL4 in different tumors in the TIMER database. ^*^P < 0.05, ^**^P  < 0.01, ^***^P  < 0.001, ^****^P  < 0.0001, ns (*P* ≥ 0.05).

TTLL4 and TUBA1B were the only two genes involved in the carboxy-terminal PTMs of tubulin that exhibited high expression in HCC and were associated with poor prognosis in the Cancer Genome Atlas (TCGA) dataset [Fig. 1(a)]. Simultaneously, Pearson correlation analysis identified the top five genes most correlated with TTLL4 expression in carboxy-terminal post-translational modifications of tubulin. Among these, TUBA1B ranked third, with a correlation coefficient of 0.438 [Fig. 1(b)]. Notably, TTLL4 was mainly expressed in hepatocytes in the tumor microenvironment (TME) [Fig. 1(c)], highlighting its potential relevance in HCC. Consistently, TTLL4 expression was higher in HCC samples than in normal tissues across multiple datasets, including TCGA [Fig. 1(d)], GSE14520 [Fig. 1(e)], GSE45436 [Fig. 1(f)], GSE54236 [Fig. 1(g)], GSE62232 [Fig. 1(h)], GSE101685 [Fig. 1(i)], and the TIMER dataset [Fig. 1(j)].

### Somatic mutational profiles and immune cell infiltration landscape of TTLL4 in HCC

**FIG. 2.**
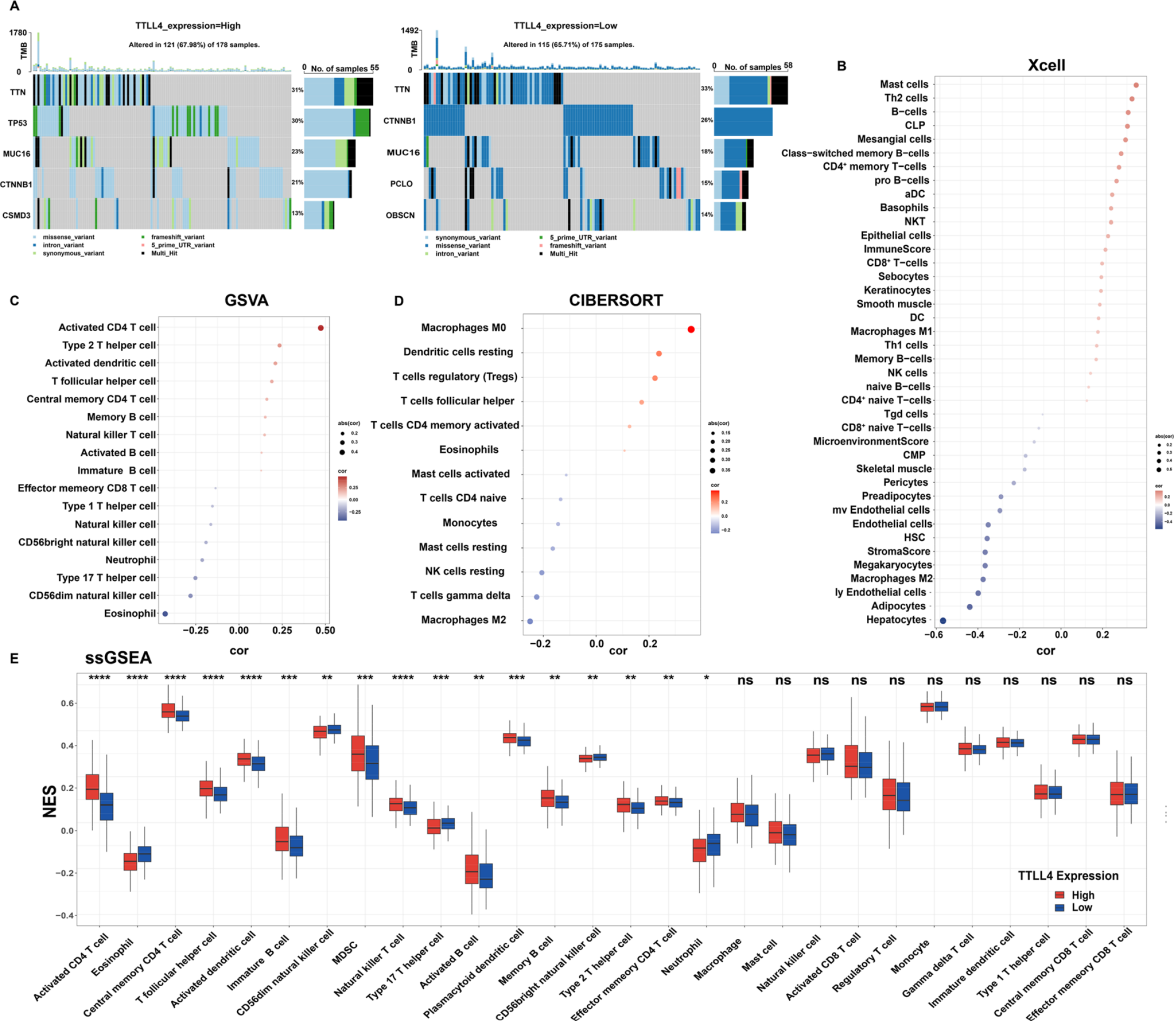
Somatic mutations and tumor-infiltrating immune cells in different expression groups of TTLL4 in the TCGA cohort. (a) Somatic mutations in different expression groups of TTLL4 in the TCGA cohort. (b) Tumor-infiltrating immune cells in different expression groups of TTLL4 in the TCGA cohort, analyzed via Xcell. (c) Tumor-infiltrating immune cells in different expression groups of TTLL4 in the TCGA cohort, analyzed via GSVA. (d) Tumor-infiltrating immune cells in different expression groups of TTLL4 in the TCGA cohort, analyzed via CIBERSORT. (e) Tumor-infiltrating immune cells in different expression groups of TTLL4 in the TCGA cohort, analyzed via ssGSEA. ^*^P < 0.05, ^**^P  < 0.01, ^***^P  < 0.001, ^****^P  < 0.0001, ns (*P* ≥ 0.05).

To assess whether TTLL4 expression affects somatic mutations, mutation maps of the two groups were generated. With respect to the mutated genes in HCC, the group with higher TTLL4 expression had higher mutation rates of TP53 (30%), MUC16 (23%), and CSMD3 (13%) than did the lower TTLL4 expression group [Fig. 2(a)].

The Xcell, GSVA, CIBERSORT, and ssGSEA algorithms were employed to examine the associations between TTLL4 expression and the infiltration levels of various immune cell types. Xcell analysis revealed correlations between TTLL4 and both mast cells and type 2 T helper (Th2) cells [Fig. 2(b)]. GSVA demonstrated that TTLL4 was associated with both activated CD4^+^ T cells and Th2 cells [Fig. 2(c)]. The CIBERSORT results revealed a correlation between TTLL4 and both macrophages and resting dendritic cells [Fig. 2(d)]. Additionally, ssGSEA revealed an association between TTLL4 and both activated CD4^+^ T cells and eosinophils [Fig. 2(e)].

### The impact of TTLL4 expression on prognosis in the TCGA cohort and clinical tissue microarrays

**FIG. 3.**
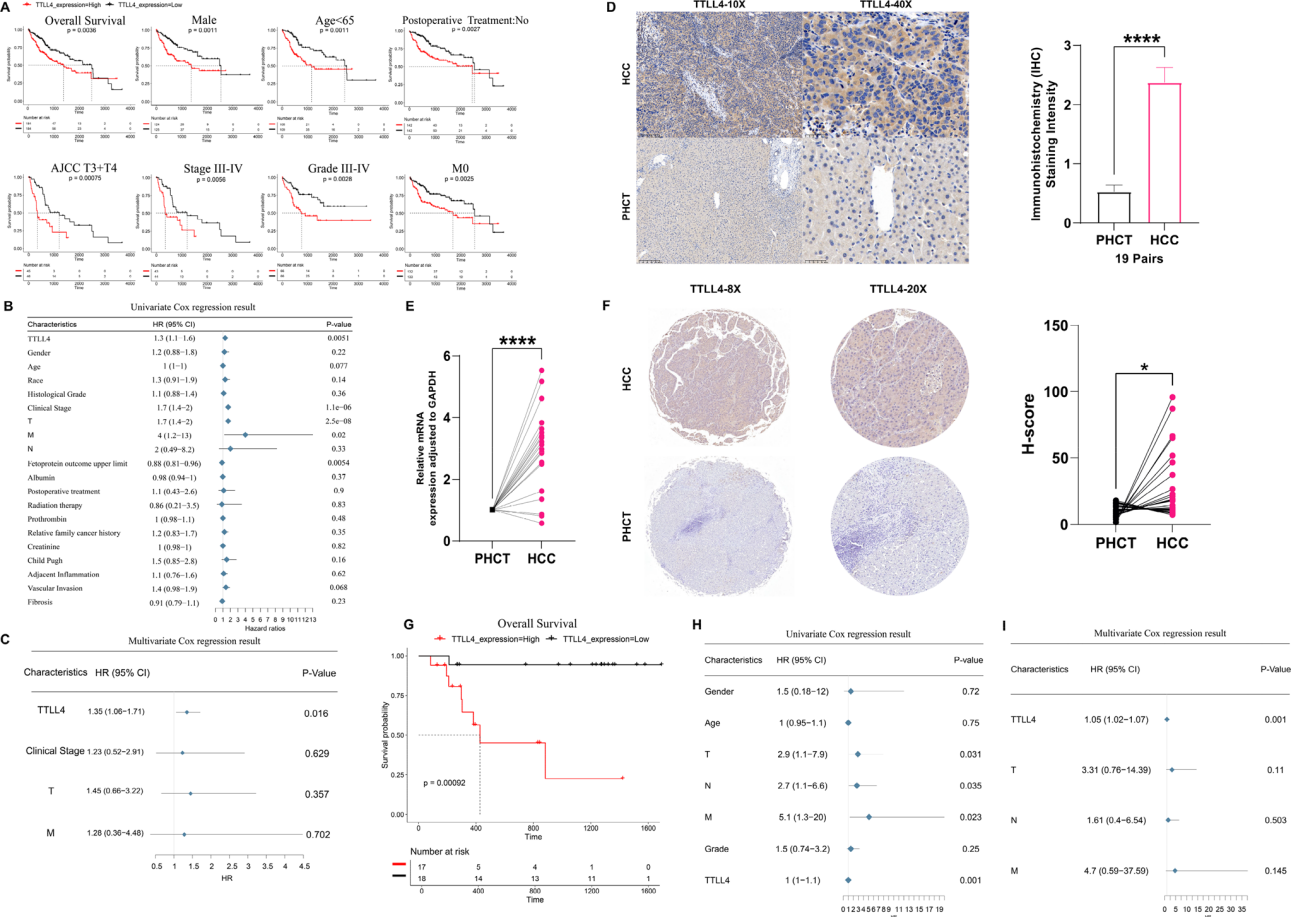
The impact of TTLL4 expression on prognosis in the TCGA cohort and clinical tissue microarrays. (a) Kaplan–Meier survival curves stratified by TTLL4 expression levels across different subgroups in the TCGA dataset. (b) Univariate Cox regression analysis of TTLL4 expression in the TCGA dataset. (c) Multivariate Cox regression analysis of TTLL4 expression in the TCGA dataset. (d) The expression of TTLL4 in clinical samples measured by IHC. (e) The expression of TTLL4 in clinical samples measured by qRT-PCR. (f) The expression of TTLL4 in clinical tissue microarrays measured by IHC. (g) Kaplan–Meier survival curves stratified by TTLL4 expression levels in tissue microarrays. (h) Univariate Cox regression analysis of TTLL4 expression in tissue microarrays. (i) Multivariate Cox regression analysis of TTLL4 expression in tissue microarrays. ^*^P  < 0.05, ^**^P  < 0.01, ^***^P  < 0.001, ^****^P  < 0.0001, ns (*P* ≥ 0.05).

To assess the prognostic value of TTLL4 in HCC, patients were divided into two groups according to the median expression of TTLL4. Kaplan–Meier analysis indicated that patients with high TTLL4 expression had lower overall survival [Fig. 3(a)]. Furthermore, in the group of males, age <65, AJCC T3 + T4, stages III–IV, grades III–IV, M0, and no postoperative treatment, high expression of TTLL4 is associated with poor prognosis [Fig. 3(a)]. Additionally, Cox regression analysis revealed that the expression level of TTLL4 was an independent prognostic factor in patients with HCC [Figs. 3(b) and 3(c)]. Formalin-fixed, paraffin-embedded sections of 19 tumor-adjacent tissue pairs were subjected to hematoxylin and eosin (H&E) and immunohistochemistry (IHC) staining. These analyses demonstrated that TTLL4 expression was predominantly localized in the cytoplasm, with markedly higher expression in tumor tissues than in their adjacent noncancerous counterparts [Fig. 3(d)]. RNA was then extracted from these 19 paired samples for qRT-PCR analysis, which confirmed that TTLL4 expression was notably elevated in tumor tissues. In three patients, TTLL4 expression in liver tumor tissues was more than fourfold higher than in normal tissues [Fig. 3(e)]. Consistently, in tissue microarrays, TTLL4 expression was higher in liver cancer tissues than in adjacent noncancerous tissues [Fig. 3(f)], correlating with poor prognosis and suggesting its potential as an independent prognostic factor [Figs. 3(g)–3(i)].

### Impact of TTLL4 knockdown on cellular function

**FIG. 4.**
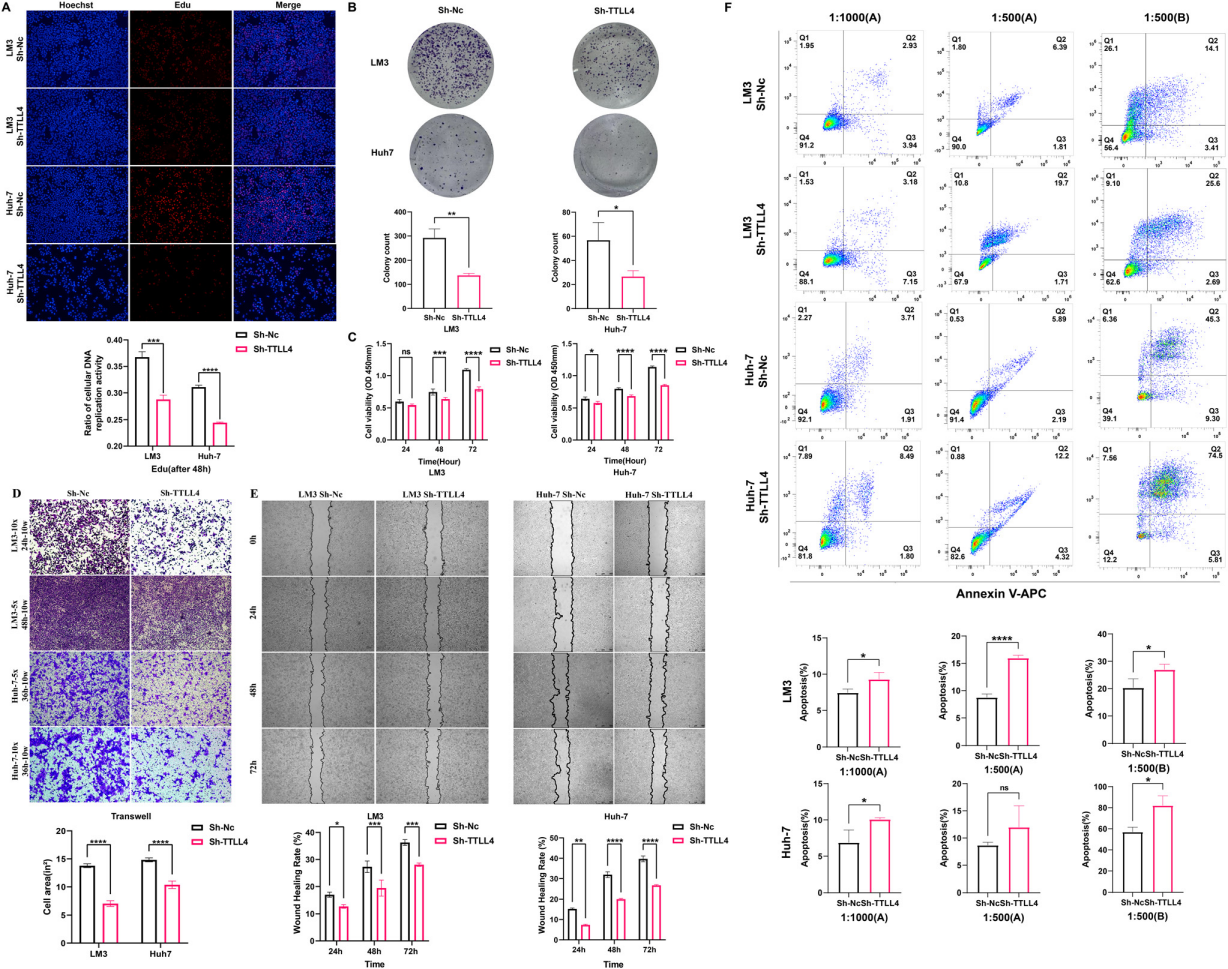
The effect of TTLL4 on proliferation, colony formation, migration, and apoptosis in HCC cells. (a) The proliferation capacity of Sh-TTLL4 HCC cells measured by EdU. (b) The effect of Sh-TTLL4 on the colony formation of HCC cells assessed by the plate clone assay. (c) The proliferation capacity of Sh-TTLL4 HCC cells assessed by CCK-8. (d) The migration capacity of Sh-TTLL4 HCC cells assessed by Transwell assay. (e) The migration capacity of Sh-TTLL4 HCC cells assessed by wound healing assay. (f) The apoptotic capacity of Sh-TTLL4 HCC cells under apoptotic inducer treatment. ^*^P  < 0.05, ^**^P  < 0.01, ^***^P  < 0.001, ^****^P  < 0.0001, ns (*P* ≥ 0.05).

EdU assays revealed that the Sh-TTLL4 group significantly inhibited the growth and proliferation of HCC cells [Fig. 4(a)]. Colony formation assays revealed that the LM3 cells formed more colonies than the Huh-7 cells did. Compared with the control cells, the TTLL4-knockdown Huh-7 cells formed larger colonies. In both the LM3 and Huh-7 cell lines, the number of clones formed in the Sh-Nc group was significantly higher than that observed in the Sh-TTLL4 group [Fig. 4(b)]. In the CCK-8 assay, no significant differences were observed in the LM3 cells at 24 h after knockdown; however, notable differences were observed at 48 and 72 h in both the LM3 and Huh-7 cells [Fig. 4(c)]. In the Transwell assay, TTLL4 knockdown reduced the ability of tumor cells to migrate through an 8 *μ*m polycarbonate membrane, with the effect being more pronounced in the LM3 cells [Fig. 4(d)]. Wound healing assays further demonstrated that TTLL4 knockdown resulted in a slower wound closure rate in both the Huh-7 and LM3 cells. After 72 h, the wound closure area in the TTLL4-knockdown group was reduced by 8.2% in the LM3 cells and 13.0% in the Huh-7 cells compared with that in the control groups [Fig. 4(e)].

### Effects of TTLL4 knockdown on the induction of apoptosis

The flow cytometry results revealed that reagent B had a significantly stronger apoptotic induction effect than did reagent A, particularly in Huh-7 cells. At a 1:500 dilution of reagent B, the percentages of apoptotic LM3 and Huh-7 cells in the Sh-TTLL4 group were 26.96% and 82.13%, respectively, while they were 20.33% and 57.07%, respectively, in the Sh-Nc group. These findings suggested that TTLL4 knockdown markedly enhanced apoptosis in tumor cells upon exposure to apoptotic agents [Fig. 4(f)]. In contrast, apoptosis rates in Huh-7 cells showed no significant variation between the Sh-TTLL4 and the Sh-Nc groups at a 1:500 dilution of reagent A. However, at a 1:1000 dilution, significant differences in apoptosis rates were observed between the Sh-TTLL4 and Sh-Nc groups in both the LM3 and the Huh-7 cells [Fig. 4(f)].

### TTLL4 expression accelerates lung metastasis in nude mice

**FIG. 5.**
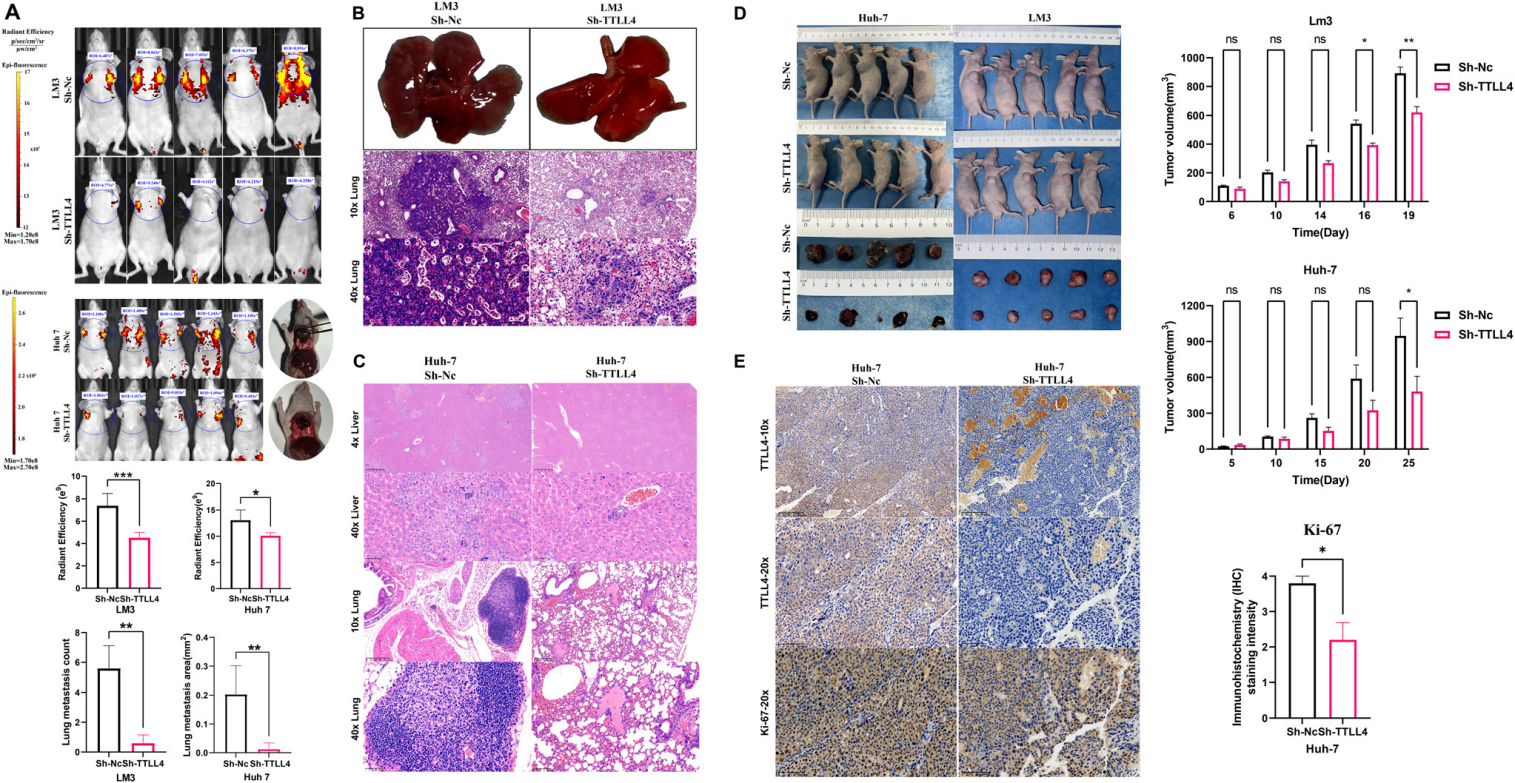
The effect of TTLL4 on subcutaneous tumorigenesis and lung metastasis of HCC cells. (a) The lung metastatic ability of Sh-TTLL4 HCC cells assessed by *in vivo* imaging. (b) The lung metastatic ability of Sh-TTLL4 LM3 cells analyzed by H&E staining of lung tissue sections. (c) The orthotopic liver colonization and lung metastatic ability of Sh-TTLL4 Huh-7 cells analyzed by H&E staining of lung tissue sections. (d) The subcutaneous tumorigenic ability of Sh-TTLL4 HCC cells. (e) The effect of TTLL4 knockdown on the expression of Ki-67 in subcutaneous tumors by IHC. ^*^P  < 0.05, ^**^P  < 0.01, ^***^P  < 0.001, ^****^P  < 0.0001, ns (*P* ≥ 0.05).

Owing to the presence of a GFP tag on the plasmid, lung metastases were visualized via live imaging of small animals. Under the same fluorescence threshold, the lung fluorescence intensity in the Sh-Nc group was markedly higher compared to that in the Sh-TTLL4 group [Fig. 5(a)]. Following lung tissue excision, distinct metastatic tumor foci were observed, which were pathologically confirmed by fixation, embedding, sectioning, and H&E staining. Additionally, the Sh-Nc group had a higher number of lung metastatic foci compared to the Sh-TTLL4 group [Figs. 5(b) and 5(c)]. Moreover, in the Sh-Nc group of Huh-7 cells, several nude mice presented visible tumor lesions in the liver, as confirmed by both visual observation and H&E staining of tissue sections. In contrast, no such lesions were observed in the Sh-TTLL4 group [Figs. 5(a) and 5(c)].

### TTLL4 knockdown inhibits subcutaneous tumor progression in HCC cells

As opposed to the control group, the TTLL4-knockdown group exhibited slower tumor growth and reduced tumor volume, with statistically significant differences observed on day 25 in the Huh-7 cell group and on days 16 and 19 in the LM3 cell group [Fig. 5(d)]. Subcutaneous tumor sections were subjected to immunohistochemical analysis. The results indicated a marked decrease in the cytoplasmic TTLL4 protein level in the Sh-TTLL4 group [Fig. 5(e)]. Ki-67, a nucleus-localized proliferation marker, is widely used to assess cell proliferation. Despite some background interference, a notable increase in the number of Ki-67-positive nuclei was observed in the Sh-Nc group. These findings suggested that TTLL4 knockdown reduced Ki-67 protein expression in tumor cells [Fig. 5(e)].

### Functional analysis

**FIG. 6.**
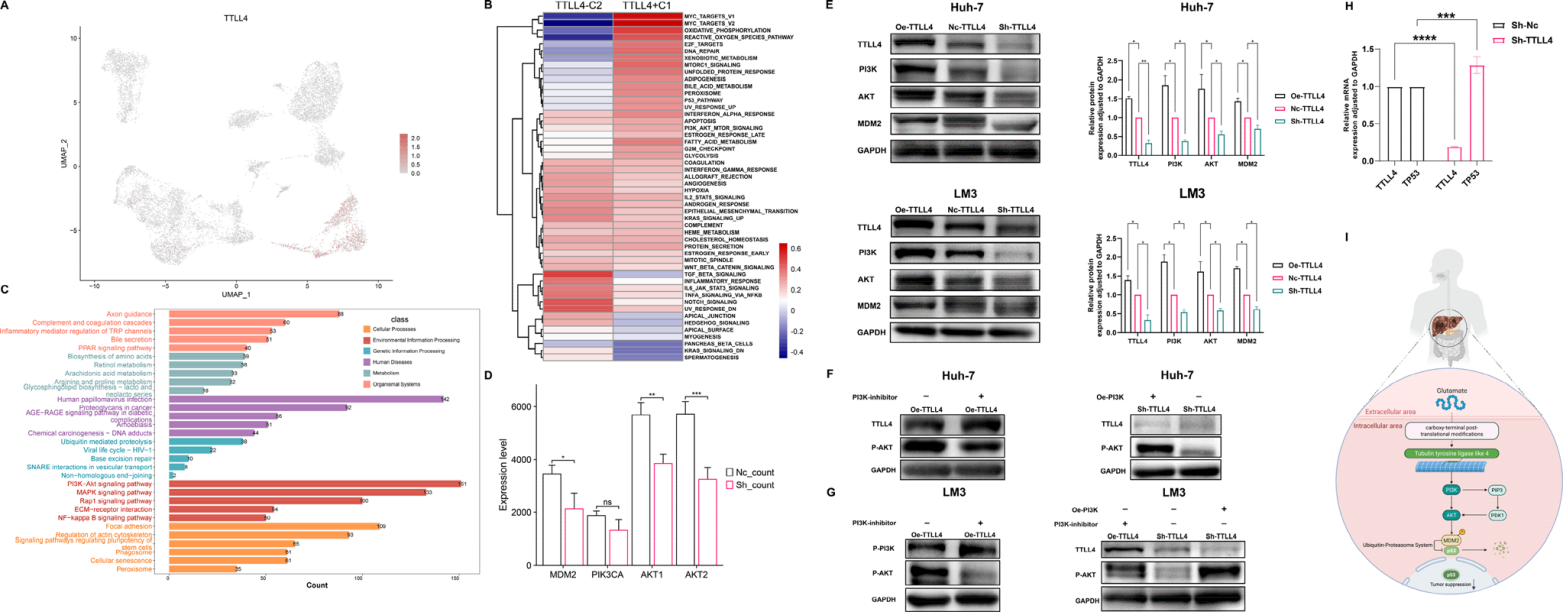
Functional analysis of TTLL4. (a) The expression of TTLL4 in hepatocytes from the GSE149614 dataset. (b) Functional analysis of TTLL4 in the GSE149614 via GSEA. (c) Functional analysis of TTLL4 in sequencing results via KEGG. (d) The effect of TTLL4 knockdown on the PI3K/AKT/MDM2 pathway proteins in sequencing results. (e) The effects of TTLL4 knockdown and overexpression on the PI3K/AKT/MDM2 pathway proteins assessed by WB. (f) The effects of PI3K inhibitors and PI3K overexpression on TTLL4-mediated regulation of the PI3K/AKT/MDM2 pathway in the Huh-7 cells by WB. (g) The effects of PI3K inhibitors and PI3K overexpression on TTLL4-mediated regulation of the PI3K/AKT/MDM2 pathway in the LM3 cells by WB. (h) The effect of TTLL4 knockdown on TP53 expression measured by qRT-PCR. (i) TTLL4's role in the PI3K/AKT/MDM2 pathway illustrated through a cellular pathway diagram. ^*^P  < 0.05, ^**^P  < 0.01, ^***^P  < 0.001, ^****^P  < 0.0001, ns (*P* ≥ 0.05).

We reported the analysis of TTLL4 expression in hepatocytes at the single-cell level [Fig. 6(a)], which were classified into subgroups C1 and C2 on the basis of TTLL4 expression levels. Functional analysis was performed on the basis of the differentially expressed genes (DEGs) between the two subgroups. GSEA also revealed that TTLL4 was highly involved in the MYC targets V1/V2, E2F targets, and PI3K–Akt signaling pathways [Fig. 6(b)]. These findings suggested that the PI3K–Akt signaling pathway may be involved in the TTLL4-mediated progression of HCC. Consistently, DEGs related to TTLL4 expression were identified on the basis of the sequencing results, and KEGG analysis was performed. The results of environmental information processing revealed that the pathway with the highest level of activation was the PI3K–AKT signaling pathway, which suggested that TTLL4 may be involved in tumorigenesis via this process. Additionally, functional enrichment analyses indicated that TTLL4 might be associated with the mitogen-activated protein kinase (MAPK) pathway, COVID-19, and focal adhesion [Fig. 6(c)]. In support of this, the knockdown of TTLL4 leads to a simultaneous decrease in the expression of MDM2, AKT1, and AKT2, although the downregulation of PIK3CA expression does not show a statistically significant difference [Fig. 6(d)].

### TTLL4 may regulate the PI3K/AKT/MDM2 pathway

Considering the results of the functional analysis of TTLL4, we explored the potential mechanisms of TTLL4. The overexpression of TTLL4 led to the upregulation of PI3K, AKT, and MDM2, whereas the downregulation of TTLL4 resulted in decreased expression of these proteins, suggesting that TTLL4 may promote the proliferation and survival of HCC cells through the PI3K/AKT/MDM2 pathway [Fig. 6(e)]. Rescue experiments demonstrated that PI3K inhibitors reduced the expression of Oe-TTLL4-induced p-AKT, while PI3K overexpression reversed Sh-TTLL4-mediated p-AKT reduction, indicating TTLL4 modulates the PI3K–AKT–MDM2 pathway via PI3K regulation [Figs. 6(f) and 6(g)]. The qRT-PCR results indicated that, in the Sh-TTLL4 groups of Huh-7 cells, TP53 expression was increased [Fig. 6(h)]. This may be because MDM2 is an upstream regulator of TP53 expression in HCC.^13,14^ Knocking down TTLL4 may reduce MDM2 levels, thereby increasing the expression of p53 and potentially suppressing tumor initiation and progression. To elucidate the pathway role of TTLL4 in HCC, a cellular pathway diagram was constructed [Fig. 6(i)].

## DISCUSSION

In recent years, advancements in the understanding of HCC mechanisms have revolutionized the therapeutic landscape for this malignancy.^15,16^ Combination therapies for HCC have demonstrated significant potential for improving patient outcomes in both neoadjuvant and systemic treatment settings.^17^ For example, the phase III IMbrave 150 study revealed that compared with sorafenib, combination first-line therapy for advanced HCC yields a significantly superior mOS (19.2 vs 13.4 months, p < 0.001).^18^ Moreover, this regimen has shown promise in conversion therapy, facilitating tumor shrinkage, necrosis, and downstaging, thereby enabling surgical intervention for patients previously deemed inoperable.^18,19^ Nevertheless, there remains a paucity of treatment strategies capable of providing long-term substantial improvements in the prognosis of patients with HCC. Therefore, elucidating the molecular mechanisms underlying HCC progression is of paramount importance.

TTLL4 is a key enzyme involved in the carboxy-terminal PTMs of tubulin, which are associated with tumor progression.^24^ Among genes related to carboxy-terminal PTMs of tubulin, TUBA1B is highly expressed and closely linked to immune cell infiltration and the expression of immune-related genes in HCC.^20,21^ Specifically, elevated TUBA1B expression leads to a reduction in CD56^+^ NK cells.^22^ Furthermore, TUBA1B is strongly associated with telomerase reverse transcriptase (TERT) mutations, the most common genetic alterations in HCC.^23^ Notably, TUBA1B and TTLL4 are the only two genes in this pathway that are highly expressed and associated with poor prognosis in the TCGA liver cancer dataset, with TTLL4 exhibiting a higher hazard ratio than TUBA1B. Additionally, Pearson correlation analysis revealed a high correlation between TTLL4 and TUBA1B expression in HCC. Based on these findings, we propose that TTLL4 and TUBA1B may play a potential synergistic role in HCC, although further experimental validation is required. Moreover, the upregulation of TTLL4 in breast cancer cells has been linked to the formation of brain metastases, modulating the interactions between breast cancer and vascular endothelial cells.^10^ Given these findings, investigating the role of TTLL4 in HCC, a vascular tumor, is critically important,^10^ as TTLL4 may represent a key molecule in promoting HCC progression. In our study, analysis of multiple cohorts revealed that TTLL4 was significantly overexpressed in HCC tissues compared with normal tissues. Furthermore, high expression of TTLL4 was associated with poor clinical outcomes in HCC patients and served as an independent prognostic indicator. These findings underscored the potential significance of TTLL4 in HCC pathogenesis and its utility as a biomarker of patient outcomes.

Within the TME, we observed elevated TTLL4 expression in HCC cells, which was closely associated with the infiltration of immune cells, particularly CD4^+^ T cells and Th2 cells. In the context of antitumor immune responses, CD4^+^ T cells play crucial roles not only in activating CD8^+^ cytotoxic T lymphocytes (CTLs) but also in producing cytokines and chemokines that indirectly contribute to antitumor immunity.^25,26^ Th1 cell-mediated cellular immunity can directly eliminate tumor cells, whereas Th2 cells and their associated cytokines mediate humoral immunity and participate in antitumor immune responses.^27,28^ However, a high proportion of Th2 infiltration has been demonstrated to increase IL-4 levels in basophil-enriched tumor-draining lymph nodes, which is correlated with poor tumor prognosis.^29,30^ Additionally, hepatitis B virus (HBV) infection is one of the pivotal factors in the causation of HCC, and the immune response triggered by its infection is considered a potential initiating factor in the progression from HBV to HCC.^31^ In this process, Th2 cells primarily secrete pro-inflammatory cytokines such as IL-4 and IL-10, resulting in persistent chronic inflammation and HBV infection.^31^ This triggers a cycle of hepatocyte damage and regeneration, along with upregulation of inflammation and fibrosis, ultimately driving the development of HCC.^31^ In our study, TTLL4 overexpression was significantly positively correlated with CD4^+^ T cells and Th2 cells, suggesting that TTLL4 may be involved in the regulation of antitumor immunity as well as the progression of HBV to HCC. This finding warrants further investigation to elucidate the precise mechanism underlying this relationship.

Our findings demonstrated that the inhibition of TTLL4 expression significantly suppressed the proliferation, colony formation, and migration of HCC cells, thereby attenuating the malignant biological behavior of HCC. Furthermore, TTLL4 inhibition markedly enhanced tumor cell apoptosis induced by proapoptotic agents, suggesting that targeting TTLL4 may potentiate the efficacy of existing HCC therapies, such as transarterial chemoembolization (TACE) and lenvatinib,^32^ thus suggesting the potential for the development of novel combination treatment strategies. In animal studies, TTLL4 knockdown effectively inhibited the origin and metastasis of HCC cells, resulting in significant reductions in tumor volume and metastatic burden.

To elucidate the potential mechanisms by which TTLL4 exerts its functions in HCC, we performed sequencing and discovered that TTLL4 may be involved in the PI3K–AKT signaling pathway via functional analysis. Moreover, the sequencing results indicated that the knockdown of TTLL4 led to a decrease in the levels of AKT1, AKT2, and MDM2 within this pathway. Although the reduction of PI3K in the Sh-TTLL4 group was not statistically significant at the RNA level, TTLL4 overexpression increased the expression of proteins in the PI3K/AKT/MDM2 pathway at the protein level, whereas TTLL4 inhibition led to a decrease in the expression of these proteins. Moreover, both PI3K inhibitors and PI3K overexpression reversed TTLL4-mediated effects on the PI3K–AKT–MDM2 axis, suggesting TTLL4 regulates this pathway through PI3K expression modulation. Activation of the PI3K/AKT signaling pathway is a crucial risk factor for early recurrence and poor prognosis in patients with HCC, and it also serves as a key determinant of cancer cell dependence on glycolytic pathways for energy production.^33^ In hepatocytes, AKT1 and AKT2, but not AKT3, are predominantly expressed.^34^ Increased expression of AKT1 and AKT2 in HCC is associated with poor clinical outcomes and plays a critical role in regulating cellular proliferation, invasion, and metabolic control.^35,36^ TP53, which encodes protein p53, will lose its tumor-suppressing function after mutations. Under normal circumstances, the p53 protein is strictly regulated by the negative regulators MDM2 and MDMX, which control the ubiquitin-mediated degradation of p53.^37,38^ In clinical trials, MDM2 inhibitors effectively dissociate the p53/MDM2 complex, inhibit MDM2-mediated ubiquitination and degradation of p53, and induce the activation and accumulation of wild-type p53 in tumor cells.^39^ Although qRT-PCR results showed an increase in TP53 expression in the Sh-TTLL4 group, further experiments are needed to verify whether TTLL4 can influence P53 expression by mediating the PI3K/AKT/MDM2 pathway. Collectively, these results provided strong evidence that TTLL4 was a potential therapeutic target for HCC.

In conclusion, TTLL4 plays a critical role in tumorigenesis by primarily mediating the polyglutamylation of tubulin in the process of PTMs.^7^ In breast cancer, TTLL4-mediated polyglutamylation of microtubules disrupts exosome homeostasis by regulating the transport of multivesicular bodies (MVBs), thereby promoting the formation of breast cancer brain metastases.^10^ In pancreatic cancer, TTLL4 facilitates cancer progression by mediating the polyglutamylation of the oncogenic scaffold protein PELP1.^11^ In HCC, TTLL4 expression was markedly upregulated and exhibited a strong correlation with patients' prognosis. The overexpression of TTLL4 potentially activates the PI3K–AKT–MDM2 pathway by regulating PI3K expression through protein polyglutamylation, thereby enhancing the malignant biological behaviors of HCC, including proliferation, migration, tumorigenesis, and metastatic capabilities. Although further experimental validation is required to identify the specific proteins and their polyglutamylation levels involved, these findings provide initial insights into the mechanistic role of TTLL4 in HCC.

## METHODS

### Data resources and descriptions

The RNA sequencing data, somatic mutation profiles, and clinical and pathological characteristics were obtained from TCGA and Gene Expression Omnibus (GEO) databases. Additionally, gene expression data across different tumor types were obtained from the TIMER 2.0 database.

Full RNA-seq data and clinical information, including expression patterns, prognostic information, somatic mutation profiles, and the immune cell infiltration landscape, were used for further analysis.

### Functional analysis

After Huh-7 cells were transfected with lentivirus, the total mRNA from the samples was extracted and enriched. Subsequently, library construction and quality control were performed, followed by sequencing (Illumina NovaSeq 6000 PE150). To elucidate the putative function of TTLL4, we performed a KEGG pathway analysis to identify the biological pathways associated with TTLL4 expression. Simultaneously, we stratified all samples into two groups on the basis of the median expression level of TTLL4 mRNA, which was obtained from public databases, to analyze the correlation between TTLL4 expression and the tumor immune microenvironment via Xcell, CIBERSORT, GSVA, and ssGSEA analyses.

### Quantitative reverse transcription polymerase chain reaction

The HCCLM3 and Huh-7 cell lines were obtained from the Cell Bank of the Chinese Academy of Sciences (Shanghai, China). Total RNA was extracted from the tissue samples and liver cancer cell lines via TRIzol reagent (Takara Biotechnology, Japan) following the manufacturer's guidelines. cDNA was synthesized via a reverse transcription kit (EnzyArtisan, China) for subsequent PCR. qRT-PCR was performed using 2× S6 Universal SYBR qPCR Mix (EnzyArtisan, China). Relative mRNA expression levels were normalized to those of GAPDH and calculated via the 2^−ΔΔCt^ method. The primers designed for amplifying TTLL4 included: forward, 5′-CCCTGAAGTGCAA-3′; and reverse, 5′-TCCTTCTTGCCAAAGCGGCTCT-3′. For TP53 amplification, the primers were: forward, 5′-GGCCATCTACAAGCAGTCACAG-3′; and reverse, 5′-GTCATCCAAATACTCCACACGC-3′.

### Immunohistochemistry

The tissue samples were preserved in 10% formalin and embedded in paraffin. The sections were treated with xylene to remove paraffin and subsequently rehydrated via a graded series of ethanol. After antigen retrieval and blocking with 5% bovine serum albumin (BSA) to prevent nonspecific binding, the sections were incubated with a panel of primary antibodies specific to the target antigens. The relevant horseradish peroxidase (HRP)-conjugated secondary antibodies were applied, followed by visualization using DAB (3,3′-diaminobenzidine) chromogenic substrate. The slides were counterstained with hematoxylin, and images were captured using a light microscope to analyze the expression of the target antigens within the tissue.

### Stable lentiviral cell line construction assay

HEK293T cells were transfected with lentiviral packaging plasmids and transfer vectors containing three different sequences: Sh (shRNA for gene knockdown), Oe (overexpression construct), and Nc (negative control). Transfection was performed via a suitable reagent. After 48 h, the lentiviral supernatants were collected, filtered, and used to infect target cells with polybrene to increase the infection efficiency. Infected cells were selected with puromycin for 7–14 days to ensure stable integration of the lentiviral constructs. Surviving cells were expanded and validated by qRT-PCR or western blotting to confirm the knockdown, overexpression, or control conditions. The resulting stable cell lines were used in subsequent functional assays. Furthermore, this study employs the PI3K inhibitor (LY294002) to suppress the expression of its downstream phosphorylated Akt (p-Akt), thereby investigating the regulatory role of TTLL4 within the PI3K/AKT/MDM2 signaling axis.

### CCK-8, EdU, and colony formation assays

A CCK-8 assay (NCM Biotech, Suzhou, China) was conducted to evaluate the impact of TTLL4 on cell proliferation. Cells were plated in 96-well plates at a density of 5000 cells per well, and absorbance was measured at 450 nm after 24, 48, and 72 h. Cells were grown in 96-well plates and subsequently incubated with Cell-Light EdU Apollo 567 (RiboBio, China) for 2 h. To assess colony formation, the transfected cells were seeded in 6-well plates and cultured for 14 days. On day 14, the colonies were fixed with methanol at room temperature for 15 min and subsequently stained with 0.1% crystal violet.

### Scratch wound healing and Transwell assays

Cell migration was evaluated via wound healing and Transwell assays. For the wound healing assay, cells were grown to 90% confluence in 6-well plates. A sterile 200 *μ*l pipette tip was used to generate a scratch in the cell monolayer. After washing three times with phosphate-buffered saline (PBS), serum-free dulbecco's modified eagle medium (DMEM) was added. Wound closure was monitored and photographed at 0, 24, 48, and 72 h after scratching. For the Transwell migration assay, 600 *μ*l of DMEM supplemented with 20% fetal bovine serum (FBS) was placed in the lower chamber of the Transwell insert, while 200 *μ*l of serum-free DMEM containing cells was added to the upper chamber. After 24, 36, and 48 h of incubation, the cells were fixed with 4% paraformaldehyde. The cells attached to the bottom of the Transwell chamber membrane were stained with 0.1% crystal violet and subsequently imaged.

### Apoptosis induction and detection assay

The cells were plated in culture dishes and permitted to grow until they reached 60%–70% confluence. The apoptosis-inducing agents A (Beyotime-C0006S, Shanghai, China) and B (Beyotime-C0005, Shanghai, China) were then added to the cells, with agent A at working concentrations of 1:1000 and 1:500 (v/v), respectively, and agent B at a working concentration of 1:500 (v/v). Following the addition of these agents, the cells were incubated for an additional 12 h. After incubation, the cells were harvested and stained with an APC-7AAD apoptosis detection kit. After staining, apoptosis was assessed via flow cytometry.

### Subcutaneous tumor implantation assay

The cells were cultured to 80%–90% confluence, digested with trypsin, and resuspended in PBS at a concentration of 1 × 10^8^ cells/ml. The suspension was mixed with cold Matrigel at a 1:1 ratio and kept on ice. Male nude mice (4–6 weeks old, n = 20) were housed under specific pathogen-free (SPF) conditions for 1 week. After the axillary area was disinfected with 70% ethanol, 200 *μ*l of the cell suspension was subcutaneously injected to form a tumor. The mice were monitored postinjection, and tumor growth was assessed regularly. Once the tumors reached a certain size, they were collected and stained with H&E as well as IHC for analysis. All procedures followed the ethical guidelines.

### Lung metastasis model via a tail vein injection assay

Male nude mice aged 4–6 weeks (n = 20) were housed in an SPF facility for 1 week before the experiment. The tumor cells were cultured to 80%–90% confluence, washed with PBS, digested with trypsin, and resuspended in sterile PBS at a density of 1 × 10^7^ cells/ml. The mice were restrained to expose the tail vein, wiped with 75% ethanol, and injected with 100 *μ*l of cell suspension into the tail vein. The injections were performed slowly to avoid air bubbles. After the injection, the health and behavior of the mice were monitored. Four weeks later, the mice were anesthetized with isoflurane for imaging to assess lung metastasis and then euthanized to harvest lung tissues for further analysis.

### Western blot

For the western blot analysis, total proteins were extracted from the cells via a standardized lysis protocol. The isolated proteins were then separated by sodium dodecyl sulfate–polyacrylamide gel electrophoresis and transferred to polyvinylidene fluoride (PVDF) membranes. The membranes were subsequently incubated overnight at 4 °C with primary antibodies specific to the target proteins. Following thorough washing, horseradish peroxidase-conjugated secondary antibodies were applied. Protein bands were visualized via an enhanced chemiluminescence (ECL) detection system and quantified with image analysis software.

### Statistical analysis

Data processing and statistical analyses were conducted via R software (version 4.0.2) and GraphPad Prism 9. Appropriate statistical methods were selected on the basis of the data characteristics. Statistical significance was defined as ^*^P < 0.05, ^**^P < 0.01, ^***^P < 0.001, ^****^P < 0.0001, and ns (p ≥ 0.05).

## Data Availability

The data that support the findings of this study are available from the corresponding author upon reasonable request.
